# Modifiable risk factors for peritoneal dialysis-related infections - a population-based cohort study on risk factors and outcomes in South Sweden

**DOI:** 10.3389/fneph.2025.1583675

**Published:** 2025-09-10

**Authors:** Oskar Ljungquist, Marta Tobijaszewska, Gustav Torisson, Giedre Martus, Mårten Segelmark, Jonas Tverring

**Affiliations:** ^1^ Division of Infection Medicine, Department of Clinical Sciences, Lund University, Lund, Sweden; ^2^ Department of Infectious Diseases, Helsingborg Hospital, Helsingborg, Sweden; ^3^ Clinical Infection Medicine, Department of Translational medicine, Faculty of Medicine, Lund University, Malmö, Sweden; ^4^ Department of Infectious Diseases, Skåne University Hospital, Malmö, Sweden; ^5^ Department of Endocrinology, Nephrology and Rheumatology, Skåne University Hospital, Lund, Sweden; ^6^ Division of Nephrology, Department of Clinical Sciences, Lund University, Lund, Sweden

**Keywords:** chronic kidney failure, peritoneal dialysis, bacterial infections, mortality, obesity

## Abstract

**Background:**

The risk of infection-related death is high in patients undergoing dialysis. This study aimed to identify the modifiable risk factors for PD-related infections in patients undergoing peritoneal dialysis.

**Methods:**

This was a population-based retrospective cohort study conducted in Skåne, South Sweden, which included all patients receiving peritoneal dialysis (PD) between 2011 and 2020. The primary outcome was PD-related peritonitis, and the secondary outcome was a composite of PD-related infections, that is, peritonitis, exit site, or tunnel infections. Time-to-event frailty models, unadjusted and adjusted for age at PD start, sex and Charleson comorbidity index, were used to investigate potentially modifiable risk factors for PD-related infections. Cox regression models were subsequently used to analyze the relationship between PD-related infection episodes and all-cause mortality during the study period.

**Results:**

In total, 545 patients were included in the study, of whom 212 (39%) patients had at least one episode of peritonitis during a median follow-up time of 1.6 years. We found that BMI ≥ 30 may be associated with a clinically relevant increased risk for PD-related infection (aHR 1.45, 95% CI 1.08-1.93, *p*-value 0.012, *n_events_* = 486), but not for peritonitis alone (adjusted Hazard Ratio, aHR, 1.34, 95% CI 0.95- 1.91; *p* = 0.099; *n_events_* = 365). Patients with >3 peritonitis episodes had an almost three-fold increased risk of all-cause mortality (aHR, 2.66; 95% CI 1.56-4.52, *p* < 0.001).

**Conclusion:**

We found that a BMI ≥ 30 may be a modifiable risk factor for peritoneal dialysis-related infections and that multiple episodes of infectious complications of peritoneal dialysis are associated with increased all-cause mortality.

## Introduction

For individuals on peritoneal dialysis, the risk of infection-related death is more than 20 times greater than that in the general population (1). Peritonitis is a serious complication in patients undergoing peritoneal dialysis (PD). A catheter inside the peritoneum, which is linked to outside containers, predisposes patients to intra-abdominal and systemic infections. Several studies have investigated the risk factors for peritonitis in PD, with somewhat divergent results. This might be explained by discrepancies in the included populations, settings, statistical methodologies, and definitions used (2–4). Previously reported risk factors for developing peritonitis include poor infection prevention care, exit-site infection, low serum albumin, higher Charlson comorbidity index (CCI) score, and lower clearance rate of urea nitrogen (2–4). Earlier studies have shown that peritonitis is associated with all-cause mortality in patients undergoing PD, and that this association gradually increases after two years of dialysis (5, 6). In our previous research, we explored the etiology and incidence of peritonitis in patients undergoing PD (7). This study aimed to investigate modifiable risk factors for peritonitis and other PD-related infections in patients receiving peritoneal dialysis in Skåne, South Sweden.

## Methods

### Study design and setting

This was a population-based retrospective cohort study in Skåne, South Sweden, including all patients who received PD between January 1^st^, 2011, and December 31^st^, 2020. Skåne had a population of close to 1.4 million people. All patients, both adults and pediatric patients, in Skåne with non-assisted PD received regular training focused on infection prevention by healthcare professionals specializing in the care of dialysis patients. Assisted PD was performed by trained staff from home care services. The region of Skåne has ten hospitals, but peritoneal dialysis is concentrated to five centers: Malmö, Lund, Helsingborg, Hässleholm, and Ystad. Only incident cases during the study were included in the study.

### Outcomes and definitions

The primary outcome measure was PD-related peritonitis. The secondary outcomes included a composite outcome of PD-related infections, including peritonitis, exit-site infection, or tunnel infection. We also aimed to report recurrent and relapsing peritonitis during the study period. Peritoneal dialysis-related peritonitis, exit-site infection, tunnel infection, pre-PD peritonitis, and recurrent and relapsing peritonitis were defined according to the International Society for Peritoneal Dialysis (8, 9). As all included patients had chronic kidney disease, a modified version of the Charlson comorbidity scale (modified CCI) was used without points assigned for chronic kidney disease. Recurrent peritonitis was defined as a peritonitis episode that occurs within four weeks of completion of therapy for a prior episode but with a different organism (9). Relapse peritonitis was defined as a peritonitis episode that occurs within four weeks of completion of therapy of a prior episode with the same organism or one sterile (culture-negative) episode (i.e., specific organism followed by the same organism, culture-negative followed by a specific organism, or specific organism followed by culture-negative) (9). Pre-PD peritonitis was defined as a peritonitis episode occurring after PD catheter insertion and prior to the commencement of PD treatment. *Clostridioides difficile* infection was defined as symptomatic diarrhea with culture-positive *C. difficile* and presence of toxins.

### Data sources and variables

Data on all patients with PD in Skåne were accessed through the Swedish Renal Registry (SNR). Microbiological data were retrieved from the Department of Clinical Microbiology, Skåne University Hospital, Lund as described previously (7). Medical records were reviewed using Melior (Siemens Healthcare Services, Upplands Väsby, Sweden). All the medical records were reviewed according to a predefined study protocol (7). Age, etiology of kidney disease, modified CCI, smoking status (previous or current smoking), and body mass index (BMI) at PD initiation were noted. Assisted PD was defined as someone other than a patient managing the PD. The PD systems were Baxter, Fresenius, or Gambro. The PD type was either continuous ambulatory peritoneal dialysis (CAPD) or automated peritoneal dialysis (APD). Data on all-cause mortality during the study period were obtained from a regional registry.

### Statistical analysis

To investigate the risk factors for peritonitis (primary outcome) and PD-related infections (secondary outcome) in peritoneal dialysis, and to account for more than one event per individual, a time-to-event frailty model was used (10) unadjusted and adjusted for age at PD start, sex and Charleson comorbidity index. In this study, only patients initiating peritoneal dialysis after January 1^st^, 2011, and before December 31^st^ 2020, were eligible. In patients with multiple PD accounts during the study period (i.e., switching from peritoneal dialysis to hemodialysis and then back to peritoneal dialysis), only the first peritoneal dialysis episode was used in the model. We focused on modifiable risk factors (smoking, BMI, and assisted PD) in the adjusted multivariate frailty model, expressed as hazard ratios with 95% confidence intervals. To investigate the timing of infection development, Kaplan-Meier estimates of the first episode of peritonitis and PD-related infections were used and stratified into different BMI groups. To investigate whether peritonitis or PD-related infections were associated with all-cause mortality during the study period, a Cox regression model was constructed, including time-varying covariates for the number of episodes of peritonitis or PD-related infections, including time-varying adjustment for BMI, smoking, diabetes mellitus, assisted PD, age, sex, and modified CCI. Statistical *p*-value of ¾ 0.05. Statistical analyses were performed using R statistical software version 4 (R Foundation for Statistical Computing (www.r-project.org).

### Ethics and patient consent statement

This study involving human participants was reviewed and approved by the Swedish Ethical Review Authority (https://etikprovningsmyndigheten.se/en/), 14^th^ of January 2021 (DNR-2020-06524) and conducted in compliance with the ethical principles adopted in the 2013 Declaration of Helsinki and the 2016 Declaration of Taipei. The study was conducted in accordance with the local legislation and institutional requirements. The ethics committee/institutional review board waived the requirement of written informed consent for participation from the participants or the participants’ legal guardians/next of kin because to make results generalizable, and since no individual data was presented in the manuscript.

## Results

### Patient disease characteristics

In total, 545 patients were included in this study. Out Of these, 212 (39%) patients suffered from at least one episode of peritonitis during the study period (range 1-9). There were 365 peritonitis episodes among all the included patients. The duration of follow-up was 1071 person-years, with a mean follow-up of 1.97 years and a median follow-up of 1.6 years. Two hundred seventy-two (50%) patients suffered from a total of 486 episodes of either peritonitis (*n* = 365), exit-site infection (*n* = 111), or tunnel infection (*n* = 10), during the study period. The proportion of pediatric patients (*n* =12) that suffered a peritonitis during the study period was 33%. The median BMI of all PD patients at PD initiation was 26.4 (range 15.1-44.5), that is, the majority of patients were overweight or obese (**Table 1**). Forty-one percent of the included patients were diagnosed with diabetes mellitus. Of the patients with peritonitis, 56 patients (26%) were on assisted peritoneal dialysis.

**Table 1:** Baseline characteristics of included patients.

Variable	n = 545	Missing (%)
Age*, years, median (range)	66 (0, 90)	0.0
Male sex	377 (69.2)	0.0
CCI modified^**^ (ref: 0)	213 (39.1)	0.0
1	67 (12.3)	0.0
2	141 (25.9)	0.0
3+	124 (22.8)	0.0
BMI*, median (range)	26.4 (15.1, 44.5)	2.8
<25	198 (37.4)	
(25-30)	195 (36.8)	
≥30	137 (25.8)	
Smoking	296 (60.0)	9.5
Etiology of kidney disease, n (%)		0.0
Acute Kidney Injury	11 (2.0)	
Congenital anomalies	2 (0.4)	
Glomerular disease	30 (5.5)	
Mixed etiology	18 (3.3)	
Nephritic glomerular disease	46 (8.4)	
Nephrotic glomerular disease	160 (29.4)	
Obstructive nephropathy	9 (1.7)	
Other	9 (1.7)	
Prerenal disease	6 (1.1)	
Renovascular disease	85 (15.6)	
Tubulointerstitial disease	69 (12.7)	
Unknown etiology	88 (16.1)	
Vasculitis	12 (2.2)	
Self-managed PD	375 (70.9)	2.9
PD type (CAPD)	343 (66.7)	5.7
PD system (Fresenius/Gambro)	284 (61.2)	14.9
Clinic		
Lund	169 (31.0)	0.0
Malmö	161 (29.5)	0.0
Other	215 (39.4)	0.0
Years with PD, median (range)	1.6 (0.1, 9.7)	0.0

*at PD commencement. ^**^CCI at PD commencement with no points given to chronic kidney disease. CCI, Charleson comorbidity index; BMI, body mass index; CAPD, continuous ambulatory peritoneal dialysis; APD, automated peritoneal dialysis.

### Analysis of risk factors for the primary outcome (peritonitis)

**Figure 1:**
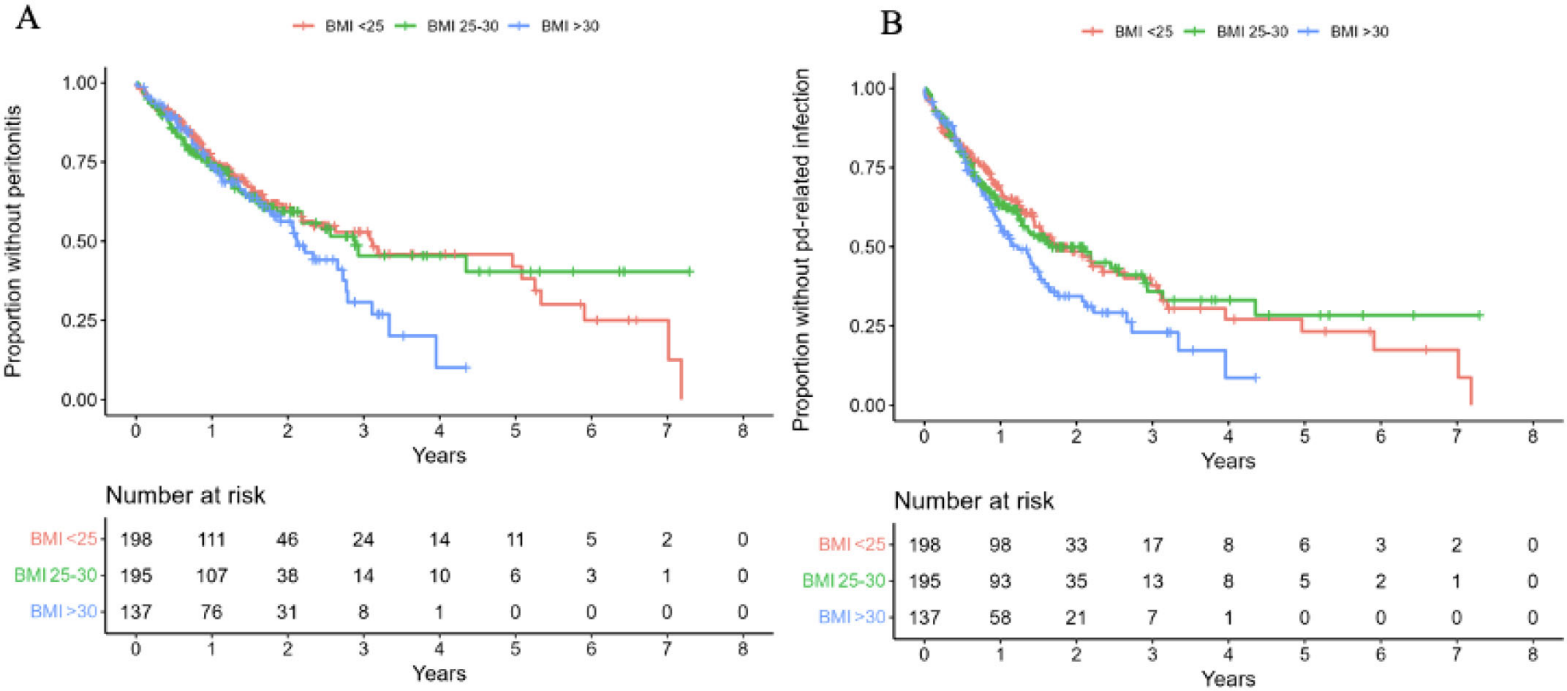
Adjusted survival curve of the primary **(A)** and secondary endpoint **(B)**.

In the unadjusted analysis, none of the pre-defined modifiable risk factors (smoking, assisted PD, or BMI) were statistically significantly associated with peritonitis. Similarly, no statistically significant associations were observed in the multivariable model adjusted for age at PD initiation, sex, modified CCI, diabetes mellitus, and the three modifiable risk factors (**Table 2**). A Kaplan-Meier estimate of the first episode of peritonitis, stratified by BMI, revealed that there was no clear difference in the timing of peritonitis development during the first two years after starting peritoneal dialysis for the different BMI groups (**Figure 1**). A modified Charlson comorbidity score of ≥ 3 (HR 1.44, 95% CI 1.02-2.04, *p* = 0.038; **Table 2**) was associated with peritonitis in the unadjusted model.

**Table 2:** Variables associated with peritonitis.

Unadjusted model^***^			Adjusted model^****^	
Variable	HR (95% CI HR)	*P*-value	HR (95% CI)	*P*-value
Age^*^ years	1.00 (0.99; 1.01)	0.597		
Male sex	0.94 (0.70; 1.27)	0.697		
CCI modified^**^ (ref: 0)	1			
1	0.78 (0.49; 1.24)	0.292		
2	1.18 (0.84; 1.66)	0.348		
3+	1.44 (1.02; 2.04)	**0.038**		
BMI (ref: <25)	1		**1**	
(25-30)	1.16 (0.83; 1.61)	0.379	1.17 (0.85; 1.62)	0.341
≥30	1.37 (0.97; 1.95)	0.078	1.34 (0.95; 1.91)	0.099
Smoking	1.19 (0.88; 1.61)	0.249	1.12 (0.83; 1.53)	0.452
Assisted PD	1.07 (0.79; 1.44)	0.678	0.97 (0.70; 1.33)	0.841
PD type CAPD (ref: APD)	1.01 (0.75; 1.36)	0.949		
PD system Baxter (ref: Fresenius/Gambro)	1.29 (0.94; 1.77)	0.112		
Clinic (ref: Lund)	1			
Malmö	1.03 (0.72; 1.46)	0.878		
Other	1.02 (0.73; 1.42)	0.921		

^*^age at PD commencement. ^***^ The unadjusted model is a frailty model. *n_events_* = 365 × at PD initiation ^**^CCI modified is Charlson comorbidity index at PD commencement with no points given to chronic kidney disease. ^****^The adjusted model is a frailty model adjusted for age at PD start, sex and CCI modified. CCI, Charlson comorbidity index; BMI, body mass index; APD, automated peritoneal dialysis; CAPD, continuous ambulatory peritoneal dialysis.

Bold values = p value < 0.05.

### Analysis of risk factors for the secondary outcome (i.e., peritonitis, exit-site or tunnel infections)

BMI ≥ 30 (HR 1.47 95% 1.10- 1.95, *p* = 0.009, *n_events_* = 486) and the Malmö site (HR 1.39 (95% CI, 1.03- 1.87, *p* = 0.03) were clinically relevant variables associated with PD-related infection in the unadjusted frailty model (**Table 3**). In the adjusted model, BMI remained associated with the composite outcome of PD-related infections (HR, 1.46; 95% CI 1.07-1.99, *p* = 0.017) to a clinically relevant degree with a moderate certainty of evidence. The other modifiable risk factors investigated in the model were not significantly associated with peritonitis, exit-site infection, or tunnel infection. A Kaplan-Meier estimate of the first episode of PD-related infection stratified by BMI revealed that patients with BMI ≥ 30 developed PD-related infections earlier after the first year of starting peritoneal dialysis compared to the lower BMI groups (**Figure 1**).

**Table 3:** Variables associated with PD-related infection.

Unadjusted model^***^			Adjusted model^****^	
Variable	HR (95% CI HR)	*P*-value	HR (95% CI)	*P*-value
Age^*^ years	1.00 (1.00; 1.01)	0.292		
Male sex	0.97 (0.75; 1.24)	0.798		
CCI modified^**^ (ref: 0)	1			
1	1.04 (0.72; 1.50)	0.845		
2	1.03 (0.77; 1.38)	0.850		
3+	1.29 (0.96; 1.73)	0.090		
BMI (ref: <25)	1		1	
(25-30)	1.05 (0.80; 1.38)	0.737	1.05 (0.80; 1.38)	0.723
≥30	1.47 (1.10; 1.95)	**0.009**	1.45 (1.08; 1.93)	**0.012**
Smoking	1.09 (0.85; 1.40)	0.482	1.05 (0.82; 1.35)	0.698
Assisted PD	1.05 (0.81; 1.35)	0.725	0.97 (0.74; 1.27)	0.827
PD type CAPD (ref: APD)	0.98 (0.76; 1.26)	0.882		
PD system Baxter (ref: Fresenius/Gambro)	1.18 (0.92; 1.52)	0.184		
Clinic (ref: Lund)	1			
Malmö	1.39 (1.03; 1.87)	**0.030**		
Other	1.25 (0.94; 1.66)	0.120		

^*^age at PD commencement. ^***^ The unadjusted model is a frailty model *n_events_* = 486 ^*^at PD initiation ^**^CCI at PD commencement with no points given to chronic kidney disease. ^***^Adjusted for age at PD start, sex and CCI ^**^No points assigned for age and chronic kidney disease.

CCI, Charlson comorbidity index; BMI, body mass index; APD, automated peritoneal dialysis; CAPD, continuous ambulatory peritoneal dialysis.

Bold values = p value < 0.05.

### Mortality

A total, 194 patients (36%) died during the study period. A Cox regression model revealed that ≥ 3 episodes of peritonitis correlated with an almost three-fold increased risk of all-cause mortality during the study period for both the unadjusted (HR 3.15, 95% CI 1.94-5.12, *p* < 0.001, *n_events_* = 194) and the model adjusted for smoking, diabetes mellitus, assisted PD, BMI groups, age at PD onset, sex, and modified CCI (HR 2.66, 95% CI 1.56-4.52, *p* < 0.001), with a high certainty of evidence. For the secondary outcome of PD-related infections, two episodes were associated with an almost two-fold increase in mortality for both the unadjusted (HR 2.14, 95% CI 1.39-3.32, *p* < 0.001) and the adjusted models (HR 2.11, 95% CI 1.10-2.01, *p* < 0.019) as was ≥ 3 episodes of PD-related infections in the unadjusted (HR 2.16, 95% CI 1.38-3.01, *p* < 0.001) and the adjusted models (HR 1.82 95% CI 1.10-3.01, *p* = 0.019) with moderate to high certainty of evidence. Assisted PD was also associated with a clinically relevant increased risk of mortality in the adjusted model with moderate certainty of evidence for both peritonitis alone (HR 1.64, 95% CI 1.17-2.23, *p* = 0.004, **Table 4**) and PD-related infections (HR 1.75, 95% CI 1.24-2.46, *p* = 0.001, **Table 5**).

**Table 4:** All-cause mortality during the study period and number of peritonitis episodes.

Unadjusted model^*^			Adjusted model^**^	
Variable	HR (95% CI HR)	*P*-value	HR (95% CI)	*P*-value
No of peritonitis episodes				
0	1		1	
1	1.24 (0.87; 1.77)	0.226	1.21 (0.83; 1.77)	0.314
2	1.77 (0.98; 3.18)	0.056	1.85 (0.99; 3.45)	0.054
≥3	3.15 (1.94; 5.12)	**<0.001**	2.66 (1.56; 4.52)	**<0.001**
BMI				
(0-25)			1	
(>25-30)			0.87 (0.61; 1.25)	0.445
≥30			0.78 (0.52; 1.17)	0.227
Smoking			0.79 (0.57; 1.09)	0.157
Diabetes mellitus			0.96 (0.64; 1.44)	0.843
Assisted PD			1.64 (1.17; 2.31)	**0.004**

*n_events_* = 194 ^*^A cox regression model unadjusted. ^**^A cox regression model adjusted for age at PD start, sex and Charleson Comorbidity Index. BMI, body mass index.

Bold values = p value < 0.05.

**Table 5:** All-cause mortality during the study period and number of episodes of PD-related infections.

Unadjusted models^*^			Adjusted models^**^	
Variable	HR (95% CI HR)	*P*-value	HR (95% CI)	*P*-value
No of PD-related infections				
0	1		1	
1	1.14 (0.79; 1.64)	0.485	1.12 (0.75; 1.65)	0.585
2	2.14 (1.39; 3.32)	**<0.001**	2.11 (1.32; 3.37)	**0.002**
≥3	2.16 (1.38; 3.38)	**<0.001**	1.82 (1.10; 3.01)	**0.019**
BMI				
(0-25)			1	
(>25-30)			0.94 (0.65; 1.35)	0.742
≥30			0.80 (0.53; 1.20)	0.273
Smoking			0.79 (0.57; 1.09)	0.150
Diabetes mellitus			0.96 (0.64; 1.44)	0.851
Assisted PD			1.75 (1.24; 2.46)	**0.001**

*n_events_* = 194 ^*^A cox regression model unadjusted. ^**^A cox regression model adjusted for age at PD start, sex and Charleson Comorbidity Index ^**^No points assigned for age and chronic kidney disease. BMI, body mass index.

Bold values = p value < 0.05.

### Relapse- and recurrence peritonitis

There were 17 (5%) and 16 (4%) episodes of relapse and recurrent peritonitis, respectively. Three patients with pre-PD peritonitis were identified in this study. The rate of infection due to *Clostridioides difficile* enteritis within three months of treatment for peritonitis was 8% (30/365). In 289 episodes (79%), the PD catheter extension/transfer set was replaced due to the peritonitis episode, and in seven episodes (2%), the catheter was replaced, and in 53 episodes (15%), peritoneal dialysis was terminated due to peritonitis.

## Discussion

### Main findings

In this 10-year, population-based cohort study including 545 individuals on peritoneal dialysis, we found that BMI ≥ 30 may be a potentially modifiable risk factor associated with a clinically relevant increase in risk for a composite endpoint of PD-related infection (i.e., peritonitis, exit-site infection, or tunnel infection) in both unadjusted and adjusted analyses with a low to moderate certainty of evidence. We also found that multiple episodes of peritonitis and PD-related infections were associated with a two to three-fold increase in all-cause mortality during the study period in unadjusted and adjusted analyses, with moderate to high certainty of evidence.

### Compared to the findings in other studies

A prospective, international study found that higher body weight, in addition to age, greater number of PD bags connected/24 h and hypoalbuminemia, was independently associated with a shorter time to first peritonitis episode (11).

Most previous studies on the risk factors for infectious complications of PD have been conducted in Asia, and they vary in methodology and statistical analyses. A large Chinese study of 1690 PD patients found that a higher BMI was associated with the first peritonitis episode and hypoalbuminemia with exit-site infection using multivariate logistic regression, not taking into account multiple events of peritonitis in the same individual (12). A smaller Chinese study (*n*=258) found that BMI, albumin, albumin/globulin ratio, CRP, and fast peritoneal solute transfer rate were independent risk factors for peritonitis in patients undergoing continuous ambulatory peritoneal dialysis using multivariate logistic regression (13). Another study conducted in China found that poor competence in exit-site care, catheter mobilization, history of catheter-pulling injury, and mechanical stress by waist belt or the protective bag of PD on the exit site were risk factors associated with exit-site infection, using a Cox proportional hazard regression analysis (4). A small Japanese study (22 outcomes in total) found an association between smoking and the risk of peritonitis during PD (14). The International Society for Peritoneal Dialysis has published guidelines for preventing peritonitis and determined that avoiding pets and hypokalemia, as well as not taking histamine-2 receptor antagonists, could be modifiable factors in reducing peritonitis episodes (9). Previous studies identified peritonitis as a risk factor for mortality in patients undergoing PD (5, 15). A Chinese study found that patients with PD peritonitis had a hazard ratio of 1.68 for mortality compared to patients without peritonitis, and the risk increased for each episode of peritonitis (16). According to a systematic review, previous studies have diverged results regarding obesity as a risk factor for mortality in PD (17).

### Our findings interpreted

We used a time-to-event frailty model in our study to analyze risk factors, which considers the time from PD initiation to peritonitis (or exit-site infection or tunnel infection) and dependency between multiple events of peritonitis in the same individual, as well as adjusting for covariates. We did not find evidence for an association between peritonitis and smoking, nor did we find that male sex and older age were associated with peritonitis, as earlier studies have suggested (18, 19). We consider our finding that a BMI ≥ 30 was associated with a 45% risk increase for the composite endpoint of PD-related infections compared with a BMI < 25 as plausible. Increased abdominal fat, with skin folds and eczema, could increase the risk of colonization and infection with skin bacteria, which could in turn spread into the PD system (20, 21). This could also be related to the challenges of surgery in obese patients, with the exit site sub-optimally placed in fat folds and reduced healing of surgical wounds (22). The difference in the timing of developing infections between BMI groups became apparent after two and one year for the primary and secondary outcomes, respectively, suggesting that an association with surgery of the PD catheter is less likely. Other factors, such as the immunological effects of obesity, could have contributed to our findings. As most of our patients were overweight or obese, weight-reduction measures might have an impact on infectious complications in our setting. Obese and non-obese PD patients use 1.5–4.25% of dextrose in their peritoneal dialysate, which is absorbed at 45% and could contribute to weight gain (17). Glucose remains the most commonly used osmotic agent in peritoneal solutions despite its well-known local and systemic adverse effects, including the risk of weight gain due to systemic glucose absorption. New generation weight reducing medications, such as glucagon-like peptide-1 agonists, with potentially beneficial effects on both diabetes and chronic kidney disease, might be important and cost beneficial for obese patients on PD to reduce antimicrobial use and infections (23).

In our clinical practice, we routinely use icodextrin, an established glucose-sparing peritoneal dialysis solution, as part of our strategy to reduce glucose exposure. This approach is particularly relevant given the potential long-term impact of glucose load on weight gain and metabolic health in PD patients.

The mortality rate in our cohort was 36% during the study period, which is slightly higher than a Chinese study (22%), which may be explained by the shorter (5-year) follow up time (24). For patients with PD, the survival rate seems to decrease each year, particularly in elderly patients (15). In our study, multiple episodes of peritonitis (≥3) and PD-related infections (≥2) were strongly associated with all-cause mortality during the study period in the unadjusted and adjusted analyses with moderate to high certainty of evidence, highlighting the clinical burden of infectious complications on peritoneal dialysis. We also found that assisted PD was associated with mortality, which is not surprising given that these patients can be expected to be more fragile and often have greater functional impairments or more severe underlying disease, rather than the increased risk being attributable to the dialysis modality itself (25). We found the rate of *Clostridioides difficile* enteritis was 8%. To the best of our knowledge, other studies have not reported this outcome previously, so it is difficult to put it into context. This should result in vigilance of abdominal pain and diarrhea in patients who have previously been treated for PD-related infections.

The observed higher infection risk at the Malmö clinic may reflect differences in patient case mix, local clinical practices, or documentation routines, but we did not have sufficient data to explore this further.

### Limitations

The strengths of our study include the population-based study design and reliance on case findings from the Swedish renal registry, which has excellent coverage of patients undergoing peritoneal dialysis. We used established definitions according to the International Society for Peritoneal Dialysis, and the frailty model allowed us to consider the time to the primary or secondary outcome as well as multiple outcome events. Limitations included only considering the first peritoneal dialysis episode in the model (only a minority of patients had multiple episodes of PD). We included only baseline measures of risk factors and comorbidities, which are often dynamic over time. We chose to focus on variables reflecting modifiable risk factors for infectious complications, but we cannot rule out other important predictors that we have overlooked, such as socio-economic and technical factors, nutritional status, residual kidney function or relevant laboratory results (such as serum albumin or serum potassium).

We did not perform a power calculation, as the sample size is individuals at risk during the study period. Although we included all PD patients in a large regional population over a decade-long period, statistical power remained limited. Several observed associations demonstrated clinically meaningful effect sizes but did not reach conventional levels of statistical significance. We acknowledge that PD patients are a heterogeneous population, and the cause of peritonitis in patients with PD is multifactorial, possibly depending on a vast number of factors. Among these, the immunological status of the host, colonization of pathological bacteria, and infection prevention measures are important, but can be difficult to measure.

### Conclusion

In patients undergoing PD, we found that a higher BMI was a potentially modifiable risk factor for peritonitis, exit-site infection, or tunnel infection. Multiple episodes of infectious complications to peritoneal dialysis were associated with all-cause mortality throughout the study period.

## Data Availability

The datasets presented in this article are not readily available because the data can be accessed after ethical approval. Requests to access the datasets should be directed to OL, oskar.ljungquist@med.lu.se.

## References

[1] Chong CH Au EH Davies CE Jaure A Howell M Lim WH. Long-term trends in infection-related mortality in adults treated with maintenance dialysis. Am J Kidney Dis. (2023) 82:597–607. doi: 10.1053/j.ajkd.2023.03.018, PMID: 37330132

[2] Ma X Shi Y Tao M Jiang X Wang Y Zang X. Analysis of risk factors and outcome in peritoneal dialysis patients with early-onset peritonitis: a multicentre, retrospective cohort study. BMJ Open. (2020) 10:e029949. doi: 10.1136/bmjopen-2019-029949, PMID: 32060152 PMC7045164

[3] van Diepen AT Tomlinson GA Jassal SV. The association between exit site infection and subsequent peritonitis among peritoneal dialysis patients. Clin J Am Soc Nephrol. (2012) 7:1266–71. doi: 10.2215/CJN.00980112, PMID: 22745277 PMC3408122

[4] Lin J Ye H Li J Qiu Y Wu H Yi C. Prevalence and risk factors of exit-site infection in incident peritoneal dialysis patients. Perit Dial Int. (2020) 40:164–70. doi: 10.1177/0896860819886965, PMID: 32072873

[5] Ye H Zhou Q Fan L Guo Q Mao H Huang F. The impact of peritoneal dialysis-related peritonitis on mortality in peritoneal dialysis patients. BMC Nephrol. (2017) 18:186. doi: 10.1186/s12882-017-0588-4, PMID: 28583107 PMC5460447

[6] Pecoits-Filho R Yabumoto FM Campos LG Moraes TP Figueiredo AE Olandoski M. Peritonitis as a risk factor for long-term cardiovascular mortality in peritoneal dialysis patients: The case of a friendly fire? Nephrol (Carlton). (2018) 23:253–8. doi: 10.1111/nep.12986, PMID: 28010053

[7] Tobijaszewska M Martus G Sunnerhagen T Segelmark M Ljungquist O. A population-based study on the incidence and aetiology of infectious complications in peritoneal dialysis in South Sweden. Infect Dis (Lond). (2024) 56:230–43. doi: 10.1080/23744235.2023.2292133, PMID: 38100541

[8] Szeto CC Li PK Johnson DW Bernardini J Dong J Figueiredo AE. ISPD catheter-related infection recommendations: 2017 update. Perit Dial Int. (2017) 37:141–54. doi: 10.3747/pdi.2016.00120, PMID: 28360365

[9] Li PK Chow KM Cho Y Fan S Figueiredo AE Harris T. ISPD peritonitis guideline recommendations: 2022 update on prevention and treatment. Perit Dial Int. (2022) 42:110–53. doi: 10.1177/08968608221080586, PMID: 35264029

[10] Amorim LD Cai J. Modelling recurrent events: a tutorial for analysis in epidemiology. Int J Epidemiol. (2015) 44:324–33. doi: 10.1093/ije/dyu222, PMID: 25501468 PMC4339761

[11] Ljungman S Jensen JE Paulsen D Petersons A Ots-Rosenberg M Saha H. Factors associated with time to first dialysis-associated peritonitis episode: Data from the Peritonitis Prevention Study (PEPS). Perit Dial Int. (2023) 43:241–51. doi: 10.1177/08968608231161179, PMID: 37021365

[12] Wu H Huang R Yi C Wu J Guo Q Zhou Q. Risk factors for early-onset peritonitis in Southern chinese peritoneal dialysis patients. Perit Dial Int. (2016) 36:640–6. doi: 10.3747/pdi.2015.00203, PMID: 27147289 PMC5174871

[13] Yin S Tang M Rao Z Chen X Zhang M Liu L. Risk factors and pathogen spectrum in continuous ambulatory peritoneal dialysis-associated peritonitis: A single center retrospective study. Med Sci Monit. (2022) 28:e937112. doi: 10.12659/MSM.937112, PMID: 35999775 PMC9422442

[14] Terada K Sumi Y Aratani S Hirama A Kashiwagi T Sakai Y. Smoking is a risk factor for endogenous peritonitis in patients undergoing peritoneal dialysis. J Nippon Med Sch. (2021) 88:461–6. doi: 10.1272/jnms.JNMS.2021_88-604, PMID: 33692295

[15] Sakacı T Ahbap E Koc Y Basturk T Ucar ZA Sınangıl A. Clinical outcomes and mortality in elderly peritoneal dialysis patients. Clinics (Sao Paulo). (2015) 70:363–8. doi: 10.6061/clinics/2015(05)10, PMID: 26039954 PMC4449459

[16] Chung MC Yu TM Wu MJ Chuang YW Muo CH Chen CH. Impact of peritoneal dialysis-related peritonitis on PD discontinuation and mortality: A population-based national cohort study. Perit Dial Int. (2022) 42:194–203. doi: 10.1177/08968608211018949, PMID: 34100316

[17] Ekart R Hojs R. Obese and diabetic patients with end-stage renal disease: Peritoneal dialysis or hemodialysis? Eur J Intern Med. (2016) 32:1–6. doi: 10.1111/nep.12986, PMID: 27067614

[18] Tian Y Xie X Xiang S Yang X Lin J Zhang X. Risk factors and outcomes of early-onset peritonitis in chinese peritoneal dialysis patients. Kidney Blood Press Res. (2017) 42:1266–76. doi: 10.1159/000485930, PMID: 29248923

[19] Wu H Ye H Huang R Yi C Wu J Yu X. Incidence and risk factors of peritoneal dialysis-related peritonitis in elderly patients: A retrospective clinical study. Perit Dial Int. (2020) 40:26–33. doi: 10.1177/0896860819879868, PMID: 32063144

[20] Ong PY Leung DY. Bacterial and viral infections in atopic dermatitis: a comprehensive review. Clin Rev Allergy Immunol. (2016) 51:329–37. doi: 10.1007/s12016-016-8548-5, PMID: 27377298

[21] Frasca D Strbo N. Effects of obesity on infections with emphasis on skin infections and wound healing. J Dermatol Skin Sci. (2022) 4:5–10. doi: 10.29245/2767-5092/2022/3.1157, PMID: 37621853 PMC10448872

[22] Tüzün Y Wolf R Engin B Keçici AS Kutlubay Z. Bacterial infections of the folds (intertriginous areas). Clin Dermatol. (2015) 33:420–8. doi: 10.1016/j.clindermatol.2015.04.003, PMID: 26051056

[23] Holliday MW Frost L Navaneethan SD. Emerging evidence for glucagon-like peptide-1 agonists in slowing chronic kidney disease progression. Curr Opin Nephrol Hypertens. (2024) 33:331–6. doi: 10.1097/MNH.0000000000000976, PMID: 38411162 PMC11126299

[24] Gu W Yi C Yu X Yang X. Metabolic syndrome and mortality in continuous ambulatory peritoneal dialysis patients: A 5-year prospective cohort study. Kidney Blood Press Res. (2019) 44:1026–35. doi: 10.1159/000502145, PMID: 31522168

[25] Rydell H Segelmark M Clyne N. Assisted peritoneal dialysis compared to in-centre hemodialysis - an observational study of outcomes from the Swedish Renal Registry. BMC Nephrol. (2024) 25:349. doi: 10.1186/s12882-024-03799-1, PMID: 39402451 PMC11475596

